# Ciliated Cell Variant of Endometrial Carcinoma in an Adenomyoma in Uterus

**DOI:** 10.7759/cureus.16148

**Published:** 2021-07-03

**Authors:** Sameera Rashid, Mohammed Akhtar

**Affiliations:** 1 Laboratory Medicine and Pathology, Hamad Medical Corporation, Doha, QAT

**Keywords:** ciliated cell variant of endometrioid adenocarcinoma, low grade endometrial carcinoma, tubal metaplasia, cilia, endometrial carcinoma

## Abstract

Ciliated cell variant of endometrioid adenocarcinoma (CCVEA) is an extremely rare tumor that has been seldom reported in the literature as low-grade endometrioid carcinoma with a favorable prognosis. CCVEA is characterized by neoplastic glands composed predominantly of ciliated cells with relatively little nuclear atypia. Recognition of the ciliated component is the key to the diagnosis of CCVEA but it can lead to diagnostic confusion with tubal metaplasia especially on endometrial biopsies. Herein, we report the case of a 56-year-old woman who presented with post-menopausal vaginal bleeding. Endometrial biopsy revealed extensive atypical complex endometrial hyperplasia composed predominantly of ciliated cells. The patient subsequently had a hysterectomy and bilateral salpingo-oophorectomy that revealed a large adenomyoma, adherent to the right ovary. The adenomyoma was extensively involved by CCVEA with some extension to the endometrial cavity. To the best of our knowledge, this is the first report of CCVEA that appears to arise in an adenomyoma.

## Introduction

Ciliated cells in the endometrium are usually seen in benign lesions especially in tubal metaplasia [1]. Scattered ciliated cells may be seen in endometrial adenocarcinoma. However, endometrial carcinomas composed predominantly of the ciliated cell are rare [2]. To date, 15 cases of ciliated cell variant of endometrial adenocarcinoma (CCVEA) have been reported in the literature [2-7]. However, in all these cases the carcinoma was arising in the endometrium. We encountered a post-menopausal female with ciliated endometrial carcinoma mainly involving a uterine adenomyoma. To the best of our knowledge, this is the first case of CCVEA with possible origin in an adenomyoma.

## Case presentation

**Figure 1:**
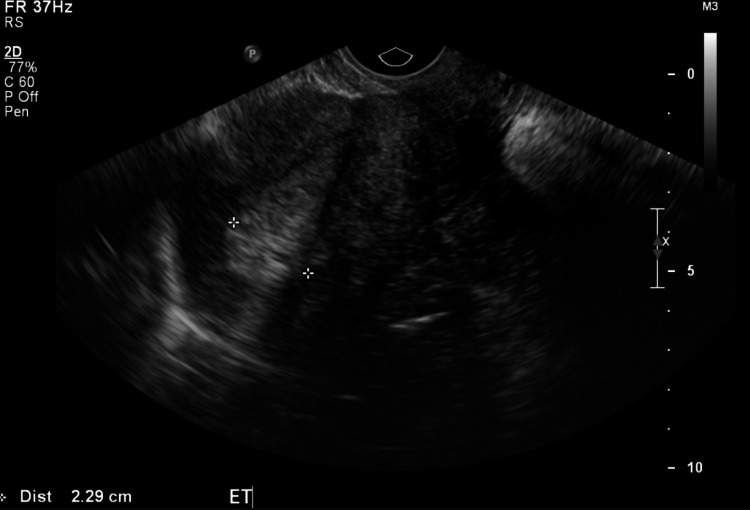
Ultrasound of the uterus showing increased endometrial thickness in the area marked by asterisks.

A 56-year-old female, multipara, eight years post-menopausal, presented with multiple episodes of vaginal bleeding. On clinical examination, the uterus appeared bulky without palpable adnexa. The uterine ultrasound showed endometrial thickening suggestive of hyperplasia (Figure 1).

**Figure 2:**
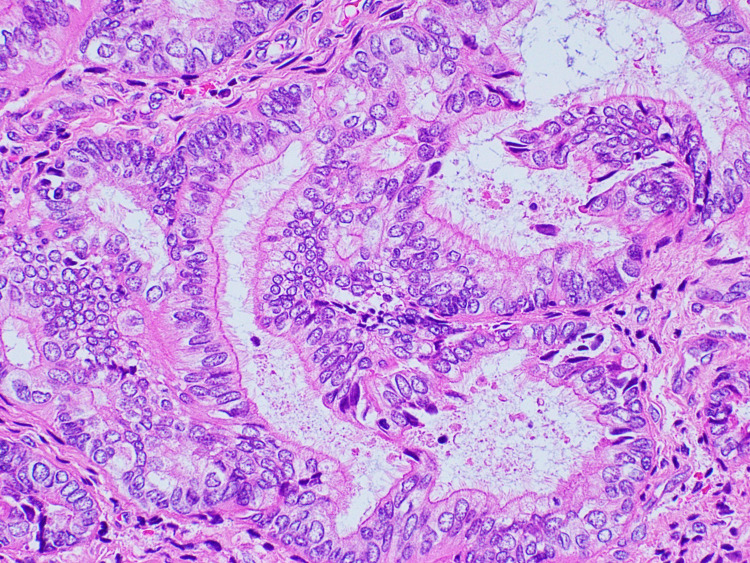
Well-differentiated endometrial adenocarcinoma showing abundant cilia.

**Figure 3:**
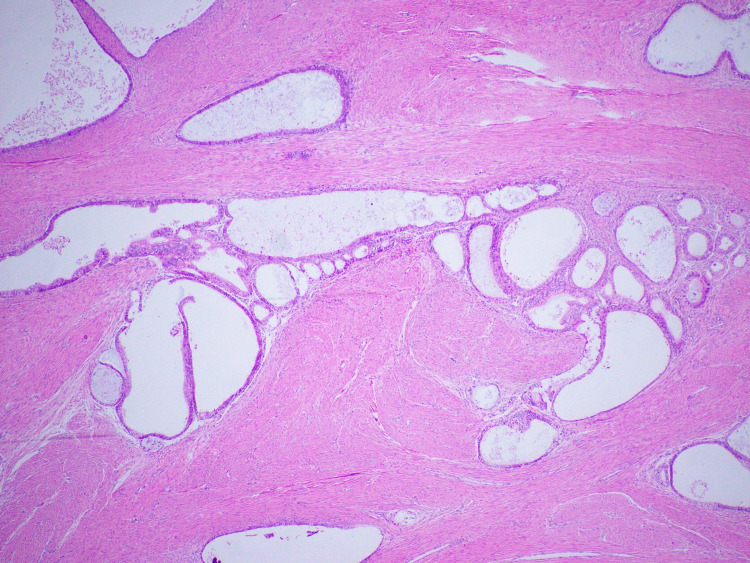
Adenomyoma with dilated endometrial glands.

**Figure 4:**
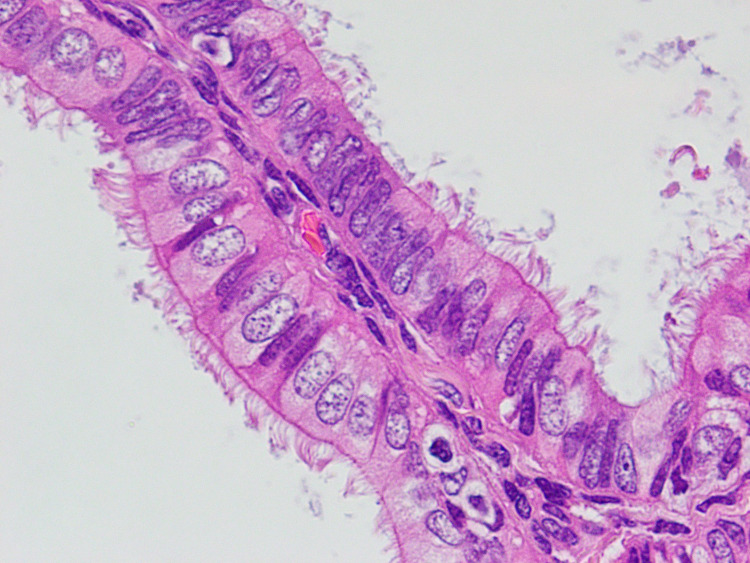
Back-to-back endometrial glands showing diffuse ciliation.

Hysteroscopy showed first-degree uterine prolapse and an endometrial polyp. Endometrial biopsy revealed extensive atypical complex endometrial hyperplasia composed predominantly of ciliated cells and carcinoma could not be ruled out. Hysterectomy and bilateral salpingo-oophorectomy were performed that grossly showed endometrial cavity expanded by thick friable material interspersed with areas of necrosis. Histologically, the endometrium was necrotic with complex atypical hyperplasia and a focal ciliated variant of endometroid carcinoma (Figure 2). The uterus also contained a solid 4 cm x 2 cm x 1.5 cm grayish tan spongy mass projecting from the uterine surface and adherent to the right ovary. This mass was composed of well-differentiated smooth muscle cells along with intricate glandular components composed of prominently ciliated cells (Figure 3). In several areas, back-to-back glands were identified with nuclear atypia indicating carcinomatous growth (Figure 4). The right ovary was free of tumor and all margins were negative. The patient was treated with adjuvant chemotherapy and radiotherapy sandwich protocol. The patient was disease-free at the time of last follow-up i.e., two years after the surgery.

## Discussion

Cilia are specialized structures associated with the epithelium of various tissues. Found in some form in all eukaryotes, these perform motility and sensory functions. Cilia are broadly divided into two types, motile and non-motile. The motile cilia are found in the lungs, respiratory tract, and middle ear. The non-motile or primary cilia play a significant role in the kidney and photoreceptors of the retina [8]. Cilia are composed of a microtubular skeleton that is surrounded by a plasma membrane. The motile cilia have a (9+2) structure with nine pairs of microtubules and a central pair as well. The primary cilia have a (9+0) structure meaning nine pairs of microtubules without any central pair. The microtubules are attached to the basal body within the cell cytoplasm [8]. Various stains can be used to identify the basal body in cilia including phosphotungstic acid-hematoxylin (PTAH) which is a variant of the trichrome stain. It also stains intracytoplasmic filaments in muscle and glial cells [9].

In many organs when adenocarcinomas arise from ciliated epithelia, the cells lose this feature of differentiation [10]. Most reported examples of ciliated adenocarcinomas are low-grade pancreatic ductal carcinoma, bronchoalveolar cell carcinoma of the lung, and borderline neoplasms of the ovary [11-13].

The Mullerian duct derivatives, cervix, endometrium, and fallopian tubes, normally contain cilia. In the endometrium, cilia are usually associated with follicular phase, anovulatory cycles, or benign proliferations like tubal metaplasia [14-15]. However, rare cases of ciliated cell variant of endometrial carcinoma have been described.

In 1983, for the first time, 10 cases of the ciliated variant of endometrial adenocarcinoma were described by Hendrickson and Kempson [2]. They did a retrospective study of 400 cases of endometrial carcinoma and 10 cases of adenocarcinoma with predominant ciliated cells (more than 75%) were identified. All these cases were of post-menopausal women, four out of 10 above were above 70 years of age and all were confined to the uterus. All 10 of the resected uteri contained residual ciliated adenocarcinoma (after biopsy), and in five cases, ciliated carcinoma invaded the myometrium. A microscopic focus of endometrioid carcinoma, thought to be a second simultaneous primary neoplasm, was present in the ovary of one of the patients.

From then on, a couple of more cases have also been reported, mostly in post-menopausal women with low-stage disease [3-7]. Hence, overall this is regarded to have a good prognosis.

There has also been a report of a case of moderately differentiated ciliated endometrioid adenocarcinoma of the endometrium (CCVEA), diagnosed by endometrial brush cytology and confirmed by histologic examination of a simultaneously obtained hysterectomy specimen [3].

Adenomyosis is a benign condition characterized by the presence of benign endometrial glands in the myometrium. Adenocarcinoma arising from adenomyoma is rare. There have been reports of endometrial carcinomas arising from adenomyoma [16] and a few reports of tumors arising from adenomyosis [17-18]. However, none of these were reported to be the ciliated variant of endometrial carcinoma. Adenomyosis may present as a polypoid and is termed polypoid adenomyoma. Atypical polypoid adenomyomas are rare lesions characterized by biphasic proliferation of complex and atypical endometrial glands within a myofibromatous stroma. Their significance lies in an increased risk of recurrence and rarely, malignant transformation into endometrioid adenocarcinoma [19]. None of these have been reported to manifest ciliated epithelium. Malignant lesions arising from adenomyoma post a higher threat of being disregarded as a fibroid/benign lesion preoperatively based on imaging studies [20].

## Conclusions

Ciliated cells in an endometrial sample are usually attributed to tubal metaplasia. However, they may be a component of the CCVEA. CCVEA is an extremely rare tumor that has been sporadically reported as a low-grade endometrioid carcinoma with a favorable prognosis. The present case, however, is a CCVEA probably arising in an adenomyoma. This report is a reminder that ciliated cells within atypical glands may represent CCVEA with malignant behavior and has the potential to metastasize. Hence, ciliated endometrium, especially in a post-menopausal woman, should be assessed with caution. Tubal metaplasia being the most common, should not impede us from looking for ciliated carcinoma.
